# Area Deprivation and Health Outcomes in Preschool Children in Japan: A Nationwide Cohort Study

**DOI:** 10.2188/jea.JE20240426

**Published:** 2025-11-05

**Authors:** Naomi Matsumoto, Etsuji Suzuki, Soshi Takao, Tomoki Nakaya, Ichiro Kawachi, Takashi Yorifuji

**Affiliations:** 1 Department of Epidemiology, Faculty of Medicine, Dentistry and Pharmaceutical Sciences, Okayama University, Okayama, Japan; 2 Graduate School of Environmental Studies, Tohoku University, Miyagi, Japan; 3 Graduate School of Science, Tohoku University, Miyagi, Japan; 4 Department of Social and Behavioral Sciences, Harvard T.H. Chan School of Public Health, Boston, United States

**Keywords:** Area Deprivation Index (ADI), child health, health inequalities, geographic clustering, outcome-wide approach

## Abstract

**Background:**

Despite Japan’s universal health insurance system, health disparities have increased since the 1990s. However, the impact of area deprivation on various aspects of child health remains understudied.

**Methods:**

This population-based cohort study followed 38,554 children born in Japan (May 10–24, 2010) from birth to age 5.5 years. Using an outcome-wide approach, Bayesian three-level logistic regression models (individuals in municipalities within eight major regions) assessed associations between municipality-level Area Deprivation Index (ADI) at birth and multiple preschool health outcomes (hospitalizations for all causes; respiratory infections; gastrointestinal diseases; Kawasaki disease; medical visits for asthma, allergic rhinitis, atopic dermatitis, food allergy, injury, intussusception; prevalence of overweight/obesity), adjusting for individual-level factors.

**Results:**

Higher ADI was associated with increased risk of all-cause hospitalization (adjusted odds ratio [aOR] per 1-standard-deviation increase in ADI, 1.04; 95% credible interval [CrI], 1.01–1.07), respiratory infections (aOR, 1.08; 95% CrI, 1.04–1.13), gastrointestinal diseases (aOR, 1.11; 95% CrI, 1.03–1.20), asthma (aOR, 1.10; 95% CrI, 1.01–1.19). Overweight/obesity at age 5.5 years also increased with higher ADI (aOR, 1.11; 95% CrI, 1.06–1.16). Higher ADI was inversely associated with Kawasaki disease (aOR, 0.86; 95% CrI, 0.77–0.96), though not robust in sensitivity analysis. Geographic clustering was observed for all outcomes, particularly at municipality level.

**Conclusion:**

We found persistent municipal-level health inequalities across various childhood health outcomes in Japan, despite its universal health insurance system. These findings suggest that policymakers should address health inequalities through comprehensive strategies targeting broader social determinants beyond health care access.

## INTRODUCTION

Studies across countries have demonstrated that health inequalities are often geographically patterned, with area-level socioeconomic status (SES) playing a crucial role beyond individual factors. Even in countries with universal health care systems such as the United Kingdom, where the National Health Service was introduced in 1946, health inequalities between areas with different levels of deprivation persist and have even widened.^1^^,^^2^ These findings highlight the importance of examining area-level SES to understand and address health disparities.

Japan has long been known for its relatively low levels of health inequality. However, health disparities across regions have increased since the mid-1990s, coinciding with economic stagnation and policy changes.^3^ Child poverty rates rose considerably during the 2000s from 13.7% in 2003 to a peak of 16.3% in 2012. Although this rate has improved to 11.5% in 2022,^4^^–^^6^ important regional variations persist, with some areas experiencing concentrated poverty that may affect children’s health outcomes. Given the well-documented spatial concentration of poverty in specific regions and neighborhoods, there is a need to examine how regional SES affects children’s health outcomes.^7^^–^^9^

The choice of Area Deprivation Index (ADI) for examining children’s health outcomes is supported by both theoretical frameworks and empirical evidence. Area-level SES influences children’s health and development through access to health care, educational resources, and recreational facilities.^10^ Environmental exposures such as air quality,^11^ housing conditions,^12^ and neighborhood safety^13^ also affect children’s physical health. Empirical studies from various countries have demonstrated the utility of area deprivation indices in child health research. In the United States, ADI has been associated with pediatric cystic fibrosis outcomes, including decreased lung function.^14^ United Kingdom studies using ADI have shown associations with young people’s consumption of foods high in fat, salt, and sugar; screen time exposure; and health knowledge.^15^ These findings highlight the relevance of ADI in capturing the broader social determinants of health that influence children’s well-being.

In Japan, prior studies have shown that ADI is associated with all-cause mortality across age groups,^16^ including children.^17^ However, evidence on how area deprivation affects other aspects of child health in Japan remains limited. This knowledge gap warrants investigation, particularly given Japan’s unique context: its universal health insurance system established in 1961, relatively homogeneous population, and distinct demographic and socioeconomic trends.^18^^–^^20^ Studies across different countries have documented associations between spatially concentrated child poverty and health outcomes^8^^–^^10^; examining these relationships in Japan’s distinct social and cultural context could provide valuable insights. Furthermore, examining SES at the municipal level provides a macro perspective that could influence broader policy-making and resource allocation, complementing micro-level insights obtained from family or neighborhood SES.^21^

The aim of this study was to examine the association between municipal-level area deprivation and child health outcomes in Japan, using a 2010 nationwide birth cohort. We used an outcome-wide approach, examining multiple health indicators simultaneously, including hospitalizations, obesity, and allergic diseases. This approach enabled identification of patterns that might be missed in single-outcome studies. Using Bayesian three-level logistic models, we also examined geographic clustering to understand spatial patterns of health inequalities across Japan.

## METHODS

### Ethical approval

This study used publicly available data from the Japanese Population Census and fully anonymized data from the Longitudinal Survey of Babies in the 21st Century, conducted by the Ministry of Health, Labour and Welfare of Japan (MHLW). Because the census data were publicly available and the longitudinal survey data were fully anonymized for secondary use, informed consent was not required. Information about the study and the opportunity to opt out was provided on our institutional website. The use of these datasets complied with all relevant ethical guidelines and data use agreements and was approved by the Institutional Review Board of the Graduate School of Biomedical Sciences, Okayama University (No. 2310-018).

### Participants and procedures

This was a population-based cohort study with secondary use of data from the Longitudinal Survey of Babies in the 21st Century, a nationwide survey conducted by the MHLW.^22^ That survey captured information on a representative sample of all infants born in Japan from May 10 to May 24, 2010, encompassing one out of every 24 births nationwide. The MHLW mailed baseline questionnaires to families of 43,767 infants at 6 months of age, achieving a response rate of 88.1% (38,554 children). The annual follow-up surveys were conducted at the infants’ age 0.5, 1.5, 2.5, 3.5, 4.5, and 5.5 years.

Survey questionnaires were designed to capture a comprehensive range of information relevant to children’s development and family situations. Key areas of inquiry encompassed children’s physical growth trajectories,^23^^,^^24^ their health records and medical experiences,^25^^,^^26^ parental educational attainment and employment status, household exposure to tobacco smoke, and various challenges and satisfaction encountered by parents in child rearing. The MHLW provided the Longitudinal Survey data linked to official birth records from Japan’s vital statistical system, which contained accurate information on birth-related variables.

### Area Deprivation Index (ADI)

Japan is administratively divided into eight major regions encompassing 47 prefectures. As of the 2010 census, these prefectures were further subdivided into 1,910 municipalities (eFigure 1).^27^ Our primary exposure measure was ADI at birth, calculated at the municipality level.

A composite indicator of geographic SES,^16^^,^^28^ ADI was derived from eight weighted variables from the 2010 Census, including the proportions of older couple households (aged 65 and above), older single-person households (aged 65 and above), rental households, single-mother households, sales and service workers, agricultural workers, blue-collar workers, and unemployed. The rationale and weighting methodology for ADI are detailed in the eMaterial 1. ADIs were initially calculated at the municipality level as absolute values and then transformed into a standardized score (in terms of standard deviation [SD]) for statistical analysis, with scores ranging from 0 (least deprived) to 1 (most deprived). This transformation was performed to facilitate comparisons across municipalities in Japan and to interpret the impact of a 1-SD change (0.633) in ADI on the outcome. Higher ADI scores have been shown to be significantly associated with all-cause mortality across different age groups, including adults and children, validating it as a measure of socioeconomic deprivation across different municipal units in Japan.^17^^,^^29^

### Child health outcomes

In this study, we selected wide outcomes from the Longitudinal Survey of Babies in the 21st Century, focusing on diseases that are either highly prevalent or have substantial effects on child health, as indicated by health care costs or concerns about long-term prognosis.^24^^,^^30^

### Preschool hospitalizations

In the Longitudinal Survey of Babies in the 21st Century, respondents were asked whether their children had been hospitalized for treatment in the past year and the cause of hospitalization. Preschool hospitalization was categorized based on the cause of hospitalization. A child was considered to have been hospitalized for a specific cause if they had at least one hospitalization for that cause during the study period (between ages 0.5 and 5.5 years). Hospitalizations owing to all causes, respiratory infections, gastrointestinal diseases (except intestinal intussusception), Kawasaki disease, and asthma were identified.

### Medical visits

A history of medical visits was also recorded based on survey responses. Children with at least one visit for asthma, allergic rhinitis, atopic dermatitis, food allergy, or injury in the preschool years were identified during the study period (age 0.5–5.5 years). The frequency of visits was not recorded in this study.

#### Other outcomes

Other outcomes included intussusception occurring before age 2.5 years and overweight or obese status at age 5.5 years. Similar to other health outcomes, this information was based on parental reports. To determine weight status, a body mass index (BMI) standard deviation score (SDS) was calculated from height and weight measurements at age 5.5 years. Using the World Health Organization (WHO) criteria, children with an SDS ≥1.5 were classified as overweight or obese in this study. To address the possibility of outliers, children with BMI SDS less than −5 at age 5.5 years (*n* = 63) were excluded from the obesity analysis.

### Statistical analysis

**Figure 1.**
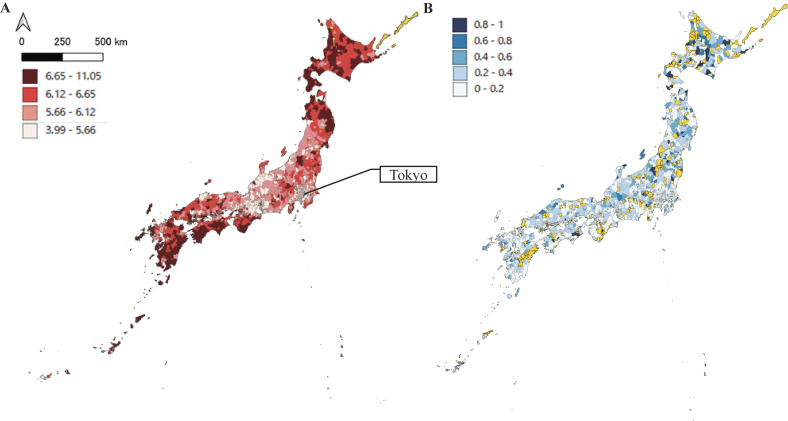
Japanese census-based Area Deprivation Index (ADI) and preschool hospitalization prevalence. Panel **A**: Area Deprivation Index (ADI). The ADI is divided into quartiles and mapped by color: brown: highest ADI (most deprived); red: third quartile; light pink: second quartile; white: lowest ADI (least deprived). Panel **B**: Preschool hospitalization prevalence. The preschool hospitalization prevalence is divided into five equal intervals from 0% to 100% and mapped using a gradient from darkest blue (highest prevalence) to white (lowest prevalence). Note: Municipalities are mapped based on the children’s birthplace, not the location of the health outcome. Municipalities with no available data are displayed in yellow.

Participants’ demographics were summarized according to administrative division at birth. To illustrate the distribution of participants, the mean and SD of the ADI and the major region (out of eight) to which participants belonged at birth are shown together with data for all Japanese municipalities for which ADI calculations were available in 2010 (eTable 1). To consider potential selection bias owing to attrition, attributes of the group lost to follow up and the analysis group are also shown. The distribution of ADIs and hospitalized proportions (regardless of the cause) of eligible children were mapped by municipality in 2010 using QGIS software (Figure 1).

Bayesian methods were chosen for analysis because of the ability to handle complex hierarchical structures in epidemiological studies using nested data structures.^31^ We performed Bayesian three-level logistic regression analyses for each outcome, with 24,140 individuals at level one, nested within 1,681 municipalities at level two, nested within eight major regions at level three. This choice allowed us to better capture the variance attributable to geographic differences in our data. To determine the most appropriate geographic hierarchy, we first conducted preliminary analyses comparing two potential level-three structures: Japan’s 47 prefectures versus its eight major regions (Hokkaido, Tohoku, Kanto, Chubu, Kinki, Chugoku, Shikoku, and Kyushu-Okinawa). Intraclass correlation coefficients for null models were calculated using both geographic divisions. The eight-region model showed stronger clustering, indicating that health outcomes were more homogeneous within major regions than within prefectures. This finding suggests that broader regional factors, such as health care resource distribution and socioeconomic conditions, may play a more important role in explaining geographic variations in child health outcomes than prefecture-level factors. We specified a Gaussian distribution for level one parameters and uniform distributions for levels two and three. We estimated the model using Markov chain Monte Carlo (MCMC) methods. We ran the MCMC algorithm for 12,500 iterations with a burn-in period of 2,500 iterations to ensure convergence. Following the crude analysis, we adjusted for the following variables obtained from birth records linked to the survey data: preterm birth (<37, ≥37 weeks; binary), low birth weight (<2,500 g, ≥2,500 g; binary), multiple birth (singleton, multiple; binary), birth order (first, second, third, or later; categorical), maternal age at birth (<30, 30–34, ≥35 years; categorical), paternal age at birth (same as maternal; categorical), and survey variables, including maternal education (bachelor’s degree or higher, vocational school/junior college graduate, high school graduate or lower; categorical), paternal education (same as maternal; categorical), maternal smoking at baseline survey (No/Yes; binary), paternal smoking at baseline survey (No/Yes; binary), and administrative division (special ward or designated city, city, town or village; categorical). Additionally, we calculated E-values for each association to assess impacts of unmeasured confounding. Larger E-values indicated stronger unmeasured confounding to shift the observed association to the null.^32^ Inspired by the perspective on multiple testing proposed by Sjölander and Vansteeland, we interpreted our results comprehensively, considering the overall pattern across all outcomes and the potential relationships between hypotheses.^33^ In this study, we used a complete case analysis approach to handle missing data. The missing data for each variable are shown in the footnote of Table 1.

**Table 1.** Participant demographics

	Birthplace distribution by administrative divisions			All

	Special ward or designated city	City	Town or village	
	(*n* = 11,008)	(*n* = 24,406)	(*n* = 3,140)	(*N* = 38,554)
Preterm birth <37 week, *n* (%)	567 (5.2%)	1,342 (5.5%)	189 (6.0%)	2,098 (5.4%)
Low birth weight <2,500 g, *n* (%)	1,047 (9.5%)	2,298 (9.4%)	297 (9.5%)	3,642 (9.4%)
Multiple birth, *n* (%)	216 (2.0%)	439 (1.8%)	68 (2.2%)	723 (1.9%)
Parity, *n* (%)				
First-born	5,692 (51.7%)	11,158 (45.7%)	1,294 (41.2%)	18,144 (47.1%)
Second born	3,915 (35.6%)	9,262 (37.9%)	1,202 (38.3%)	14,379 (37.3%)
Third or later born	1,401 (12.7%)	3,986 (16.3%)	644 (20.5%)	6,031 (15.6%)
Maternal age at birth, *n* (%)				
<30	3,676 (33.4%)	9,637 (39.5%)	1,351 (43.0%)	14,664 (38.0%)
30–34	4,221 (38.3%)	8,868 (36.3%)	1,126 (35.9%)	14,215 (36.9%)
≥35	3,111 (28.3%)	5,901 (24.2%)	663 (21.1%)	9,675 (25.1%)
Paternal age at birth, *n* (%)				
<30	2,624 (23.8%)	7,011 (28.7%)	1,009 (32.1%)	10,644 (27.6%)
30–34	3,718 (33.8%)	8,283 (33.9%)	1,056 (33.6%)	13,057 (33.9%)
≥35	4,464 (40.6%)	8,705 (35.7%)	1,029 (32.8%)	14,198 (36.8%)
Missing	202 (1.8%)	407 (1.7%)	46 (1.5%)	655 (1.7%)
Maternal educational attainment, *n* (%)				
Bachelor’s degree or higher	3,347 (30.4%)	5,024 (20.6%)	417 (13.3%)	8,788 (22.8%)
Vocational school/junior college graduate	3,761 (34.2%)	8,787 (36.0%)	1,139 (36.3%)	13,687 (35.5%)
High school graduate or below	2,508 (22.8%)	7,203 (29.5%)	1,091 (34.7%)	10,802 (28.0%)
Missing	1,392 (12.6%)	3,392 (13.9%)	493 (15.7%)	5,277 (13.7%)
Paternal educational attainment, *n* (%)				
Bachelor’s degree or higher	5,129 (47.3%)	8,431 (35.1%)	822 (26.6%)	14,382 (37.9%)
Vocational school/junior college graduate	1,608 (14.8%)	3,847 (16.0%)	540 (17.5%)	5,995 (15.8%)
High school graduate or below	2,718 (25.1%)	8,344 (34.7%)	1,236 (40.0%)	12,298 (32.4%)
Missing	1,392 (12.8%)	3,392 (14.1%)	493 (15.9%)	5,277 (13.9%)
Maternal smoking at age 6 months, *n* (%)	697 (6.3%)	1,720 (7.1%)	270 (8.6%)	2,687 (7.0%)
Non-smoking at 6 months	10,281 (93.4%)	22,617 (92.7%)	2,859 (91.1%)	35,757 (92.7%)
Smoking at 6 months	697 (6.3%)	1,720 (7.0%)	270 (8.6%)	2,687 (7.0%)
Missing	30 (0.3%)	69 (0.3%)	11 (0.4%)	110 (0.3%)
Paternal smoking at age 6 months, *n* (%)	4,145 (38.5%)	10,068 (42.3%)	1,465 (47.7%)	15,678 (41.7%)
Non-smoking at 6 months	6,621 (60.1%)	13,733 (56.3%)	1,604 (51.1%)	21,958 (57.0%)
Smoking at 6 months	4,145 (37.7%)	10,068 (41.3%)	1,465 (46.7%)	15,678 (40.7%)
Missing	242 (2.2%)	605 (2.5%)	71 (2.3%)	918 (2.4%)
Daycare use at age 1.5 years, *n* (%)	2,674 (27.7%)	5,790 (27.5%)	781 (29.5%)	9,245 (27.7%)
No daycare use at age 1.5 years	2,674 (24.3%)	5,790 (23.7%)	781 (24.9%)	9,245 (24.0%)
Daycare use at age 1.5 years	6,963 (63.3%)	15,269 (62.6%)	1,870 (59.6%)	24,102 (62.5%)
Missing	1,371 (12.5%)	3,347 (13.7%)	489 (15.6%)	5,207 (13.5%)
Birthplace distribution by major regions, *n* (%)				
Kanto	5,251 (47.7%)	7,087 (29.0%)	515 (16.4%)	12,853 (33.3%)
Hokkaido	546 (5.0%)	645 (2.6%)	239 (7.6%)	1,430 (3.7%)
Tohoku	312 (2.8%)	1,797 (7.4%)	467 (14.9%)	2,576 (6.7%)
Chubu	1,512 (13.7%)	4,793 (19.6%)	616 (19.6%)	6,921 (18.0%)
Kinki	1,963 (17.8%)	4,497 (18.4%)	373 (11.9%)	6,833 (17.7%)
Chugoku	643 (5.8%)	1,461 (6.0%)	175 (5.6%)	2,279 (5.9%)
Shikoku	0 (0.0%)	955 (3.9%)	124 (3.9%)	1,079 (2.8%)
Kyushu	781 (7.1%)	3,171 (13.0%)	631 (20.1%)	4,583 (11.9%)

We calculated median odds ratios (MORs) and 95% credible intervals (CrIs) for all models to examine geographic clustering. The MOR quantifies the variation between clusters (municipalities and major regions) by comparing individuals from randomly chosen clusters.^34^ The 80% interval odds ratio (IOR-80%) measures the impact of cluster-level covariates (eg, ADI) on unexplained between-cluster variation.^35^^,^^36^ Whereas MOR focuses on general heterogeneity between clusters, IOR-80% indicates how much of this variation is explained by ADI.^36^ An IOR-80% not containing 1 indicates that the effect of ADI is substantial relative to the residual between-cluster (municipality) variation, suggesting that differences in ADI account for parts of the between-cluster outcome variation.

Sensitivity analyses were performed only for participants who had responded to all surveys during the follow-up period. All analyses were performed using Stata SE version 18 (StataCorp., College Station, TX, USA). Statistical significance was set at *P* < 0.05, and all tests were two-tailed.

## RESULTS

### Study population characteristics

The final analytic sample comprised 24,866–38,554 children (64.5%–100% of the 38,554 initial respondents) for each outcome (eFigure 2). Table 1 presents the baseline characteristics stratified by administrative division. Rural areas had lower proportions of firstborns (special wards or designated cities vs cities vs towns or villages: 51.7% vs 45.7% vs 41.2%), younger maternal age (33.4% vs 39.5% vs 43.0%, age <30 years), lower maternal education levels (34.8% vs 23.9% vs 15.8%, bachelor’s degree or higher), and higher parental smoking rates (maternal: 6.3% vs 7.1% vs 8.6%; paternal: 38.5% vs 42.3% vs 47.7%). The demographic characteristics of participants according to municipal-level ADI quartiles are shown in eTable 2. Areas with higher deprivation (Q4) were characterized by higher proportions of third or later-born children, younger parents, lower parental educational levels, and higher parental smoking rates. These areas were more likely to be towns or villages. The proportion of missing data tended to be higher in more deprived areas. Participants lost to follow-up at age 5.5 years had higher rates of preterm birth (5.8% vs 5.2%), maternal age <30 years (46.5% vs 33.4%), maternal education less than high school (41.8% vs 28.9%), and maternal smoking (11.6% vs 4.4%) (eTable 3).

### Distribution of area deprivation

The mean ADI across all municipalities was 6.11 (SD, 0.72) (eTable 1). A spatial pattern emerged, with higher ADI values observed as areas became less urbanized, indicating a gradient from urban areas to towns/villages. Mean ADIs for special wards or designated cities, cities, and towns/villages were 5.71 (SD, 0.77), 6.05 (SD, 0.65), and 6.28 (SD, 0.73), respectively. Figure 1 and eFigure 3 illustrate the geographic distribution of ADI and preschool health outcomes.

### Associations between ADI and child health outcomes

**Figure 2.**
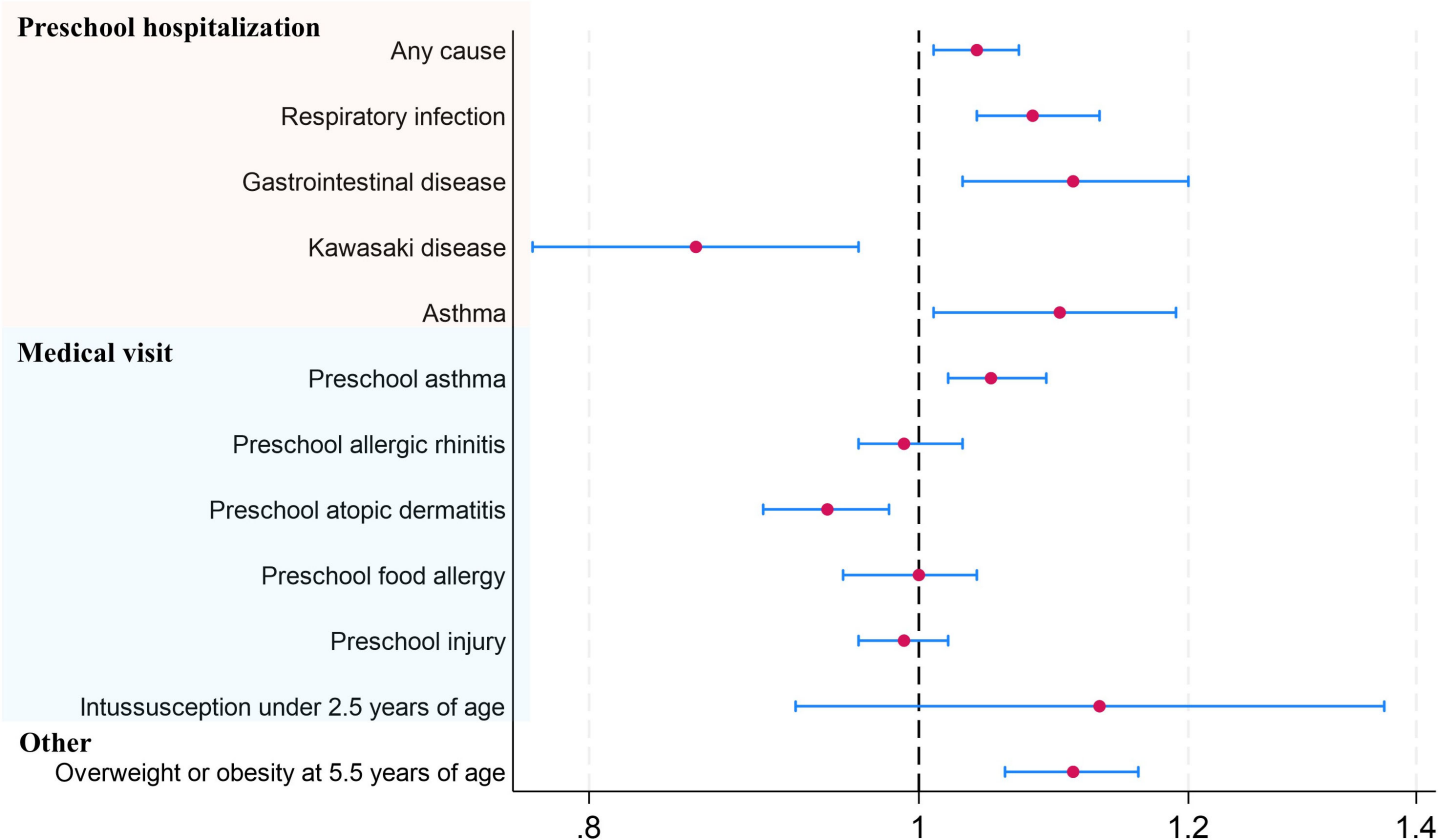
Area Deprivation Index and child health outcomes (Bayesian mixed-effects logistic three-level model). The figure presents odds ratios with 95% credible intervals (CrIs) for various child health outcomes associated with a 1-standard deviation increase in ADI. Outcomes include hospitalization (all-cause, respiratory infections, gastrointestinal diseases, Kawasaki disease, asthma), medical visits (asthma, allergic rhinitis, atopic dermatitis, food allergy, injury), intussusception, and overweight/obesity status. The model adjusts for factors such as preterm birth, low birth weight, multiple births, birth order, parental age and education, parental smoking, and administrative division.

In both crude and adjusted models, higher ADI was associated with an increased risk of preschool hospitalizations for various causes (Table 2 and Figure 2). For all-cause hospitalizations, the adjusted odds ratio (aOR) per 1-SD increase in ADI was 1.04 (95% CrI, 1.01–1.07). Specifically, respiratory infections (aOR 1.08; 95% CrI, 1.04–1.13) and gastrointestinal diseases (aOR 1.11; 95% CrI, 1.03–1.20) showed strong associations with ADI.

**Table 2.** Odds ratios for child health outcomes per 1-standard deviation increase in Area Deprivation Index

	Crude model					Adjusted model^b^				
	OR^a^	95% CrI		E-value^c^	E-value for CrI	OR^a^	95% CrI		E-value^c^	E-value for CrI
Preschool hospitalization										
Any cause	1.04	1.00	1.07	1.23	1.01	1.04	1.01	1.07	1.24	1.10
Respiratory infection	1.09	1.03	1.15	1.41	1.21	1.08	1.04	1.13	1.39	1.25
Gastrointestinal disease	1.12	1.03	1.22	1.49	1.19	1.11	1.03	1.20	1.46	1.22
Kawasaki disease	0.82	0.72	0.93	1.72	1.36	0.86	0.77	0.96	1.60	1.26
Asthma	1.10	1.01	1.21	1.44	1.09	1.10	1.01	1.19	1.42	1.12
Medical visit										
Preschool asthma	1.03	0.99	1.08	1.22	1.11	1.05	1.02	1.09	1.29	1.16
Preschool allergic rhinitis	0.97	0.94	1.01	1.20	1.14	0.99	0.96	1.03	1.08	1.24
Preschool atopic dermatitis	0.92	0.88	0.97	1.38	1.21	0.94	0.90	0.98	1.32	1.45
Preschool food allergy	0.93	0.88	0.97	1.37	1.19	1.00	0.95	1.04	1.06	1.27
Preschool injury	0.96	0.93	1.00	1.23	1.07	0.99	0.96	1.02	1.09	1.18
Intussusception under 2.5 years of age	1.19	0.90	1.52	1.66	1.45	1.13	0.92	1.37	1.51	1.40
Other										
Overweight/obesity at 5.5 years of age	1.16	1.10	1.22	1.59	1.43	1.11	1.06	1.16	1.45	1.31

CrI, credible interval; OR, odds ratio.

^a^The odds ratios are posterior median values from Bayesian analysis.

^b^Adjusted for preterm birth, low birth weight, multiple birth, birth order, mother’s age category, father’s age category, maternal education level, paternal education level, maternal smoking, paternal smoking, and municipality.

^c^E-values for CrIs were calculated from 95% credible intervals closest to the null value.

Higher ADI was also associated with increased risks of overweight/obesity at age 5.5 years (aOR 1.11; 95% CrI, 1.06–1.16). For intussusception before age 2.5 years, we observed a trend toward increased risk with higher ADI, although this association did not reach statistical significance (aOR 1.13; 95% CrI, 0.92–1.37). Conversely, higher ADI showed a protective effect against Kawasaki disease (aOR 0.86; 95% CrI, 0.77–0.96).

Among allergic diseases, a statistically significant association was observed only with medical visits for asthma and allergic rhinitis in the adjusted model (aOR for asthma: 1.05; 95% CrI, 1.02–1.09).

E-values for the observed associations are presented in Table 2, suggesting that substantial unmeasured confounding would be needed to explain the observed associations, particularly for respiratory infections and gastrointestinal diseases.

### Geographic clustering

Table 3 presents MORs at the municipality and major region levels. Geographic clustering was observed for all outcomes, especially at the municipality level, and was attenuated after adjustment, except for Kawasaki disease and allergic diseases. For all-cause hospitalization, the MOR at municipality level was 1.26 (95% CrI, 1.20–1.31) in the null model.

**Table 3.** Median odds ratios in assessing geographic clustering effects at municipal and district area levels for child health outcomes^a^

Health Outcomes	Null model			Crude model			Adjusted model^b^		
	MOR	95% CrI		MOR	95% CrI		MOR	95% CrI	
Preschool hospitalization									
Any cause									
level 2 (Municipalities)	1.26	1.20	1.31	1.25	1.20	1.30	1.26	1.21	1.32
level 3 (Major Regions)	1.34	1.16	1.72	1.34	1.14	1.80	1.28	1.13	1.54
Respiratory infection									
level 2 (Municipalities)	1.35	1.29	1.42	1.34	1.23	1.41	1.33	1.23	1.42
level 3 (Major Regions)	1.54	1.23	2.26	1.44	1.20	1.98	1.40	1.17	1.85
Gastrointestinal disease									
level 2 (Municipalities)	1.54	1.39	1.70	1.55	1.40	1.70	1.51	1.38	1.67
level 3 (Major Regions)	1.73	1.28	2.81	1.70	1.28	2.86	1.56	1.25	2.18
Kawasaki disease									
level 2 (Municipalities)	1.17	1.12	1.24	1.10	1.05	1.16	1.29	1.18	1.44
level 3 (Major Regions)	1.26	1.04	1.76	1.31	1.06	1.71	1.35	1.08	1.91
Asthma									
level 2 (Municipalities)	1.32	1.14	1.64	1.42	1.27	1.66	1.40	1.28	1.54
level 3 (Major Regions)	1.22	1.06	1.54	1.18	1.04	1.43	1.16	1.02	1.38
Medical visit									
Preschool asthma									
level 2 (Municipalities)	1.29	1.24	1.36	1.29	1.23	1.35	1.30	1.24	1.37
level 3 (Major Regions)	1.16	1.06	1.33	1.15	1.05	1.33	1.15	1.05	1.34
Preschool allergic rhinitis									
level 2 (Municipalities)	1.24	1.18	1.30	1.24	1.19	1.29	1.27	1.20	1.33
level 3 (Major Regions)	1.21	1.08	1.46	1.23	1.09	1.46	1.20	1.08	1.41
Preschool atopic dermatitis									
level 2 (Municipalities)	1.20	1.14	1.26	1.19	1.13	1.25	1.11	1.06	1.26
level 3 (Major Regions)	1.34	1.14	1.72	1.39	1.16	1.90	1.37	1.17	1.76
Preschool food allergy									
level 2 (Municipalities)	1.28	1.19	1.35	1.30	1.25	1.36	1.26	1.19	1.35
level 3 (Major Regions)	1.22	1.07	1.50	1.24	1.09	1.50	1.19	1.07	1.40
Preschool injury									
level 2 (Municipalities)	1.12	1.08	1.18	1.10	1.07	1.14	1.11	1.07	1.16
level 3 (Major Regions)	1.11	1.03	1.24	1.09	1.01	1.26	1.06	1.01	1.13
Others									
Intussusception under 2.5 years of age									
level 2 (Municipalities)	1.79	1.38	2.24	1.40	1.23	1.61	1.68	1.46	2.18
level 3 (Major Regions)	1.87	1.13	3.79	1.94	1.17	4.43	1.56	1.09	2.51
Overweight/obesity at 5.5 years of age									
level 2 (Municipalities)	1.17	1.10	1.24	1.15	1.09	1.24	1.10	1.05	1.19
level 3 (Major Regions)	1.24	1.10	1.51	1.22	1.09	1.43	1.20	1.07	1.41

CrI, credible interval; MOR, median odds ratio.

^a^Random intercepts were allowed for municipalities where a child and their parents resided at birth.

^b^Adjusted for preterm birth, low birth weight, multiple birth, birth order, mother’s age category, father’s age category, maternal education level, paternal education level, maternal smoking, paternal smoking, and municipality.

IOR-80% analysis revealed that for all examined outcomes, the IOR-80% of both crude and adjusted models included 1 (eTable 4). This suggests that while ADI showed average associations with several health outcomes, the magnitude and even direction of these associations could vary substantially across municipalities when accounting for residual between-municipality variations.

### Sensitivity analyses

In a sensitivity analysis, we restricted the sample to children with no missing responses at any time point during the follow-up period (*n* = 20,508–28,293). The results were consistent with the main analysis, where missing responses were classified as no outcome, except for Kawasaki disease (eTable 5). For Kawasaki disease (*n* = 28,204), the protective association of ADI observed in the main analysis was not evident in the sensitivity analysis (aOR 0.89; 95% CrI, 0.68–1.11). Sensitivity analyses using various combinations of covariates to address collinearity among potentially correlated variables (preterm birth, low birth weight, multiple birth, birth order) did not substantially alter the results (data available upon request).

## DISCUSSION

This study provided evidence of persistent municipal-level health inequalities among preschool children in Japan, despite the country’s long-established universal health insurance system. Our findings demonstrated that higher area deprivation is associated with increased risks of various adverse health outcomes, including hospitalizations for respiratory infections and gastrointestinal diseases, as well as higher risks of obesity, and asthma. Through an outcome-wide approach, these results address a critical knowledge gap in understanding the impact of area deprivation on child health in Japan.

Previous studies have primarily examined the association between neighborhood-level ADI and child health outcomes. At this finer geographic scale, research has shown strong associations between local area deprivation and various health outcomes. For instance, studies using ADI at the neighborhood level (10-km radius) have demonstrated the strongest associations with hospitalization rates, compared with larger geographic scales.^37^ Among children with cystic fibrosis, each decile increase in local area deprivation has been associated with worse respiratory outcomes, with those in the most deprived areas having approximately 20% greater odds of experiencing multiple pulmonary exacerbations.^14^ Studies have also found associations between neighborhood-level socioeconomic factors and various health indicators, including obesity-related outcomes.^38^^,^^39^ Whereas these neighborhood-level studies highlight the important impact of local deprivation, understanding socioeconomic disparities at broader administrative levels remains crucial. Municipal-level deprivation may exert a weaker influence on individual health outcomes owing to its larger geographic scale, yet it is a key unit for policy planning, resource allocation, and public health interventions. Some studies have examined municipal-level factors and health outcomes,^40^ but relatively few have specifically investigated the relationship between area deprivation and child health at this level. Our study extends this work by demonstrating that, despite the expected attenuation of effects, municipal-level deprivation remains significantly associated with various child health outcomes. These findings suggest that socioeconomic disparities at this broader administrative level also play an important role in shaping child health and should not be overlooked in public health planning.

Our findings are aligned with the WHO’s social determinants of health framework,^41^ showing how area-level factors influence child health through biological embedding.^42^^,^^43^ The observed geographic clustering, particularly at municipal level, emphasizes that health inequalities manifest within specific local contexts. These patterns reflect the seminal findings of the United Kingdom’s Black Report in 1980,^44^ which first documented persistent health inequalities within a universal health care system. Our study extends this discussion to Japan, a country with distinctly different cultural and social characteristics. Despite having one of the world’s longest-standing universal health insurance systems (established in 1961), Japan exhibits persistent municipal-level health disparities in child health outcomes. This suggests a universality to health inequalities that transcends geographic and cultural boundaries, and indicates that universal health care access alone may not eliminate inequalities rooted in broader socioeconomic factors.^45^

Our findings suggest several potential pathways through which area deprivation might influence child health outcomes. First, the stronger associations observed for preventable conditions, such as respiratory infections and gastrointestinal diseases, compared with other outcomes, suggest that health-related behaviors and health care-seeking patterns may play important mediating roles. Previous studies have shown that these behaviors are influenced by both individual and community-level socioeconomic factors.^46^^,^^47^ Second, geographic clustering patterns at the municipality level indicate that beyond individual characteristics, local factors play crucial roles in shaping child health outcomes. The IOR-80% analysis (eTable 4) further revealed that for all examined outcomes, the interval for the association with ADI included 1, suggesting that while average associations were present, the impact of ADI could vary across municipalities and that other local contextual factors likely contribute to the observed outcome variations. The association between area deprivation and respiratory conditions (respiratory infection and asthma) may reflect environmental inequity,^48^^,^^49^ a classic pathway of place effect where disadvantaged populations face disproportionate exposure to environmental hazards.^50^ Third, the differential associations across various health outcomes—strong for preventable conditions but weak or absent for conditions like atopic dermatitis—suggest that area deprivation may operate through specific pathways rather than affecting all health outcomes uniformly. The patterns of geographic clustering varied across outcomes, with stronger clustering for preventable conditions than for allergic diseases, further supporting this pathway-specific effect. For instance, Kawasaki disease showed an inverse association with area deprivation, though not robust in sensitivity analyses. This pattern aligns with its characteristic epidemiological profile of higher incidence in more affluent populations,^51^^,^^52^ illustrating the complex interplay between socioeconomic factors and health outcomes.

Through our outcome-wide approach,^53^^,^^54^ we uncovered diverse aspects of health inequalities that single-outcome studies might overlook. Our findings suggest several policy directions, including systematic monitoring of health inequalities and area-specific resource allocation systems that consider deprivation levels. Community-based interventions focusing on preventive care and environmental improvements in disadvantaged areas might help address these disparities. These approaches could inform policy discussions in both countries with universal health care systems and those working toward universal coverage.

Our study has several strengths, including its large, nationally representative sample, use of a comprehensive set of child health outcomes, and application of advanced statistical methods to account for geographic clustering. This study also has several limitations. The dynamic nature of area-level deprivation and potential residential mobility were not considered. Differential attrition during follow-up may have introduced selection bias. The reliance on parental reports for health outcomes may introduce reporting bias. Despite adjusting for various confounding factors and calculating E-values, substantial residual confounding likely remains and may affect our results. Additionally, we could not assess how municipal welfare policies, particularly variations in child medical subsidies, might influence the relationship between area deprivation and health outcomes. These policies could serve as either effect modifiers or mediators; for instance, more generous subsidies in highly deprived areas might attenuate the impact of deprivation on health outcomes, whereas limited subsidies might amplify existing disparities. Future research should explicitly examine these potential pathways, incorporating objective health measures, clinical diagnoses, and detailed data on local welfare policies.

Future research should address these limitations and explore the mechanisms underlying the observed associations. Longitudinal studies that follow children into adolescence and adulthood could elucidate the long-term impacts of early life exposure to area deprivation. Mixed-methods approaches and comparative studies between countries could provide valuable insights into how different social and cultural contexts shape the impact of area deprivation on child health.^55^ Additionally, studies that directly measure community health literacy levels and examine their relationship with child health outcomes and area deprivation would be valuable. Note that our findings are based on 2010 birth cohort data, the latest comprehensive national birth cohort survey. The size and scope of this dataset enabled robust three-level multilevel analysis across multiple health outcomes, providing valuable baseline evidence for understanding how area-level factors influence child health outcomes through various pathways. Although important societal changes have occurred since then—particularly the coronavirus disease 2019 pandemic, which likely exacerbated existing health inequalities^56^^,^^57^—this dataset’s comprehensive nature and methodological rigor provide crucial insights into the mechanisms of health disparities. Our estimates might be conservative compared with current conditions, highlighting the urgent need for continued monitoring of these relationships through similarly robust methodological approaches.

In conclusion, this study reveals how geographic health inequalities at the municipal level can persist even within universal health care systems, raising fundamental questions about child health equity. Our outcome-wide analysis demonstrated stronger associations between area deprivation and preventable conditions, suggesting that comprehensive community-level interventions focusing on preventive care systems may be particularly effective. Rather than targeting specific components of deprivation in isolation, policymakers should address health inequalities through integrated strategies that strengthen community resources and preventive care infrastructure in disadvantaged areas. As countries face health care reform and increasing inequality, particularly in the context of growing concerns about post-pandemic health disparities, our results provide important evidence for promoting health equity from early childhood using systematic, multi-faceted approaches that address the interconnected pathways through which area deprivation affects child health.

## SUPPLEMENTARY MATERIAL

eMaterial 1. Area deprivation index based on the Japanese census

eTable 1. Demographics of administrative divisions with study participants and administrative divisions across Japan

eTable 2. Participant characteristics by area deprivation index (ADI) quartiles at birth^a^

eTable 3. Comparison of baseline characteristics between participants who completed the 5.5-year follow-up survey and those lost to follow-up

eTable 4. Odds ratios for child health outcomes per 1-standard deviation increase of area deprivation index: sensitivity analysis using 80% interval odds ratio (IOR-80%)

eTable 5. Odds ratios for child health outcomes per 1-standard deviation increase of area deprivation index: sensitivity analysis including only participants who responded to all six longitudinal surveys

eFigure 1. Distribution of 1,910 municipalities across eight major regions of Japan

eFigure 2. Flowchart of participants

eFigure 3. Geographic distribution of multiple child health outcomes and area deprivation index across Japan
