# Erratum for “Area Deprivation and Health Outcomes in Preschool Children in Japan: A Nationwide Cohort Study”

**DOI:** 10.2188/jea.JE20250272

**Published:** 2025-11-05

**Authors:** Naomi Matsumoto, Etsuji Suzuki, Soshi Takao, Tomoki Nakaya, Ichiro Kawachi, Takashi Yorifuji

**Affiliations:** 1 Department of Epidemiology, Faculty of Medicine, Dentistry and Pharmaceutical Sciences, Okayama University, Okayama, Japan; 2 Graduate School of Environmental Studies, Tohoku University, Miyagi, Japan; 3 Graduate School of Science, Tohoku University, Miyagi, Japan; 4 Department of Social and Behavioral Sciences, Harvard T.H. Chan School of Public Health, Boston, MA, USA

There were errors in the implementation of the statistical analysis described in the accepted version of the above article [J Epidemiol, Accepted Article, doi:10.2188/jea.JE20240426].^1^ Specifically, in the Bayesian three-level logistic model, the variables for level 2 (individuals) and level 3 (major regions) were inadvertently swapped in the analysis code.

The statistical methodology described in the original article remains correct. However, correcting this implementation error led to numerical changes in the results reported. A comprehensive list of corrections is provided in the accompanying Errata, and the fully corrected versions of Table 2, Table 3, and Figure 2 are also published with this notice.

The main modifications include updates to the numerical values presented in the Abstract, Results, and all relevant tables and figures. As a result of these corrections, the previously reported association for allergic rhinitis is no longer statistically significant. Furthermore, the interpretation of the 80% interval odds ratio (IOR-80%) has been revised, as the credible intervals for all outcomes now include 1.

Despite these corrections, the main conclusions and the overall direction of the study remain unchanged. The authors sincerely apologize for these errors and any inconvenience they may have caused.

**Publisher note:**

This correction has been reflected in the printed version of the manuscript in question.

## Figures and Tables

**Table:** 

Location in Manuscript	Description of Correction
Abstract	Numerical values (adjusted odds ratios [aOR], 95% credible intervals [CrI]) for all reported outcomes were updated. The association with allergic rhinitis is no longer reported as significant.
Results (Text)	All reported numerical results (aOR, 95% CrI) were updated throughout the section. The paragraph describing the IOR-80% analysis was entirely revised to state that the credible intervals for all outcomes now include 1. The interpretation regarding the statistical significance of allergic rhinitis was removed.
Discussion (Text)	Sentences discussing the magnitude of associations, the significance of allergic rhinitis, and the interpretation of the IOR-80% analysis were revised to align with the corrected results.
Table 2	The entire table was replaced with a new version containing the corrected aORs, 95% CrIs, and E-values.
Table 3	The entire table was replaced with a new version containing the corrected median odds ratios (MORs).
Figure 2	The figure was replaced with a new version redrawn based on the corrected results.
eTable 4	The entire supplementary table for the 80% interval odds ratio (IOR-80%) analysis was replaced with a new version.
eTable 5	The entire supplementary table for the sensitivity analysis was replaced with a new version.

aOR, adjusted odds ratio; CrI, credible interval; IOR-80%, 80% interval odds ratio; MOR, median odds ratio.

## SUPPLEMENTARY MATERIAL

Area Deprivation and Health Outcomes in Preschool Children in Japan: A Nationwide Cohort Study [J Epidemiol (2025) doi:10.2188/jea.JE20240426. Epub ahead of print.]

## Errata

The reason for all corrections listed below is the same: an error in the implementation of the analysis code for the three-level logistic model described in the original article. Specifically, the level-2 (individual) and level-3 (regional) variables were inadvertently swapped. The statistical method itself remains correct. This single implementation error necessitated the correction of all results derived from the model.
