# Supplementary material for: Area Deprivation and Health Outcomes in Preschool Children in Japan: A Nationwide Cohort Study
Source: J Epidemiol. 2025 Nov 5;35(11):472–81. doi: 10.2188/jea.JE20240426 (PMC12527406; doi:10.2188/jea.JE20240426)
Supplement: Supplementary file 1 [file je-35-472-s001.pdf]

eMaterial 1. Area deprivation index based on the Japanese census

Area Deprivation Index (ADI) is a social indicator of socio-economic disadvantages of geographic areas. In adult populations, higher levels of area deprivation consistently correlate with increased mortality and cancer incidence.[1, 2] For pediatric populations, a growing body of research, particularly in the United States of America (USA), has examined this relationship across a range of health outcomes including infant mortality,[3] mental health and developmental problems,[4] oral health outcomes,[5, 6] obesity,[7] and asthma.[8] It utilizes subregions as units to reflect the composition of populations living in areas characterized by lower living standards and increased need for assistance. While primarily calculated using census data, several approaches exist for weighting individual factors.

The Japanese ADI employed in this study, developed by Nakaya et al.,[12] is based on Gordon's method[9] grounded in Townsend's concept of relative poverty.[10] It aimed to estimate the composition of economically disadvantaged households by municipality, using a composite of census indicators. The weighting of these indicators was consistent with micro-data analysis results, derived through the following procedure:

(1) Define economically disadvantaged households based on the Japanese General Social Survey (JGSS).

(2) Predict the defined disadvantaged households using a logistic regression model, with household or individual attributes as explanatory variables. The explanatory variables are limited to those for which statistical data at the municipal level are available as census indicators.

(3) The coefficients obtained from the logistic regression model serve as weights to composite the census index for each municipality, thus calculating the ADI. The ADI used in this study is defined as a weighted sum of census variables as follows:[11]

$$ADI_i = k \times (2.99 \times \textit{Proportion of elderly couple households}_i + 7.57 \times \textit{Proportion of elderly single-person households}_i + 17.4 \times \textit{Proportion of single-mother households}_i + 2.22 \times \textit{Proportion of rented houses}_i + 4.03 \times \textit{Proportion of sales and service workers}_i + 6.05 \times \textit{Proportion of agricultural workers}_i + 5.38 \times \textit{Proportion of blue-collar workers}_i + 18.3 \times \textit{Unemployment rate}_i)$$

where i is the area index (in this case, the area is municipalities). k is an arbitrary positive constant, but since it does not affect the relative ADI calculation, we used 1 in this study.

The process of deriving weights from the estimated odds ratios in the logistic regression analysis, which predicts "economically disadvantaged households" using micro-data from Japanese social surveys measuring poverty, can be considered an internal validation.[11] External validation was evidenced by the observed association between higher ADI values and increased probability of death in prospective study design.[12] Additionally, studies have shown correlations between higher ADI and increased mortality rates for various causes at the municipal level in Japan, including among children aged 0-14 years[13]. Consequently, the ADI appears to adequately reflect the degree of area deprivation in this sample, including for pediatric populations.

The 2010 census data source used to calculate the ADI in this study is available at: https://www.e-

stat.go.jp/stat-search/files?page=1&toukei=00200521&tstat=000001039448

REFERENCES

1. Stafford M, Marmot M. Neighbourhood deprivation and health: does it affect us all equally? *Int J Epidemiol*. 2003;32(3):357-366.

2. Kataoka A, Fukui K, Sato T, et al. Geographical socioeconomic inequalities in healthy life expectancy in Japan, 2010-2014: An ecological study. *Lancet Reg Health West Pac*. 2021;14:100204.

3. Yun JW, Kim YJ, Son M. Regional Deprivation Index and Socioeconomic Inequalities Related to Infant Deaths in Korea. *J Korean Med Sci*. 2016;31(4):568-578.

4. Sharifi V, Dimitropoulos G, Williams JVA, et al. Neighborhood material versus social deprivation in Canada: different patterns of associations with child and adolescent mental health problems. *Soc Psychiatry Psychiatr Epidemiol*. Published online May 5, 2024. 2025;60(4):823-836.doi:10.1007/s00127-024-02681-7

5. Blair YI, McMahon AD, Macpherson LMD. Comparison and relative utility of inequality measurements: as applied to Scotland's child dental health. *PLoS One*. 2013;8(3):e58593.

6. Lee HH, Dziak JJ, Avenetti DM, et al. Association between neighborhood disadvantage and children's oral health outcomes in urban families in the Chicago area. *Front Public Health*. 2023;11:1203523.

7. Anderson LN, Fatima T, Shah B, et al. Income and neighbourhood deprivation in relation to obesity in urban dwelling children 0-12 years of age: a cross-sectional study from 2013 to 2019. *J Epidemiol Community Health*. 2022;76(3):274-280.

8. Brokamp C, Jones MN, Duan Q, et al. Causal Mediation of Neighborhood-Level Pediatric Hospitalization Inequities. *Pediatrics*. 2024;153(4). e2023064432.doi:10.1542/peds.2023-064432

9. Gordon D. Census based deprivation indices: their weighting and validation. *J Epidemiol Community Health*. 1995;49 Suppl 2(Suppl 2):S39-44.

10. Shorrocks A, Townsend P. Poverty in the United Kingdom. A survey of household resources and standards of living. *Econ J* . 1980;90(360):954.

11. Nakaya T. Evaluating socioeconomic inequalities in cancer mortality by using areal statistics in Japan: A note on the relation between the municipal cancer mortality and the areal deprivation index. *Proceedings of the Institute of Statistical Mathematics Vol 59, No 2, 239–265 (2011) 265*. 59(2):239-265.

12. Nakaya T, Honjo K, Hanibuchi T, et al. Associations of all-cause mortality with census-based neighbourhood deprivation and population density in Japan: a multilevel survival analysis. *PLoS One*. 2014;9(6):e97802.

13. Nakaya, T. and Ito, Y. eds. The atlas of health inequalities in Japan. Springer Nature, 2019.

eTable 1. Demographics of administrative divisions with study participants and administrative divisions across Japan

	Administrative divisions with study participants				Administrative divisions across Japan			
	Special ward or designated city (n=193)	City (n=764)	Town or village (n=724)	All (n=1,681)	Special ward or designated city (n=193)	City (n=767)	Town or village (n=941)	All (n=1,901)
Areal deprivation index, mean (SD)	5.71 (0.77)	6.05 (0.65)	6.28 (0.73)	6.11 (0.72)	5.71 (0.77)	6.06 (0.66)	6.36 (0.75)	6.17 (0.74)
Major regions, n (%)								
Hokkaido	10 (5.2%)	33 (4.3%)	102 (14.1%)	145 (8.6%)	10 (5.2%)	34 (4.4%)	144 (15.3%)	188 (9.9%)
Tohoku	5 (2.6%)	74 (9.7%)	129 (17.8%)	208 (41.2%)	5 (2.6%)	74 (9.6%)	153 (16.3%)	232 (12.2%)
Kanto	67 (34.7%)	174 (22.8%)	98 (13.5%)	339 (20.2%)	67 (34.7%)	174 (22.7%)	117 (12.4%)	358 (18.8%)
Chubu	34 (17.6%)	158 (20.7%)	119 (16.4%)	311 (59.7%)	34 (17.6%)	158 (20.6%)	157 (16.7%)	349 (18.4%)
Kinki	51 (26.4%)	120 (15.7%)	77 (10.6%)	248 (74.4%)	51 (26.4%)	121 (15.8%)	102 (10.8%)	274 (14.4%)
Chugoku	12 (6.2%)	51 (6.7%)	42 (5.8%)	105 (80.7%)	12 (6.2%)	52 (6.8%)	55 (5.8%)	119 (6.3%)
Shikoku	0 (0.0%)	38 (5.0%)	40 (5.5%)	78 (85.3%)	0 (0.0%)	38 (5.0%)	57 (6.1%)	95 (5.0%)
Kyushu	14 (7.3%)	116 (15.2%)	117 (16.2%)	247 (14.7%)	14 (7.3%)	116 (15.1%)	156 (16.6%)	286 (15.0%)

SD, standard deviation.

eTable 2. Participant characteristics by area deprivation index (ADI) quartiles at birth[a]

	ADI Quartiles[b]				
	Q1 (3.99–5.66) (n=17,115)	Q2 (5.66–6.12) (n=11,521)	Q3 (6.12–6.65) (n=6,588)	Q4 (6.65–11.05) (n=3,330)	All (n=38,554)
Preterm birth <37 weeks, n (%)					
37 weeks or later	16,203 (94.7%)	10,890 (94.5%)	6,222 (94.4%)	3,134 (94.1%)	36,449 (94.5%)
22 to 36 weeks	911 (5.3%)	630 (5.5%)	364 (5.5%)	193 (5.8%)	2,098 (5.4%)
Missing	1 (0.0%)	1 (0.0%)	2 (0.0%)	3 (0.1%)	7 (0.0%)
Low birth weight <2,500 g, n (%)					
More than 2,500 g	15,544 (90.8%)	10,416 (90.4%)	5,961 (90.5%)	2,984 (89.6%)	34,905 (90.5%)
Less than 2,500 g	1,570 (9.2%)	1,104 (9.6%)	625 (9.5%)	343 (10.3%)	3,642 (9.4%)
Missing	1 (0.0%)	1 (0.0%)	2 (0.0%)	3 (0.1%)	7 (0.0%)
Multiple birth, n (%)	304 (1.8%)	255 (2.2%)	112 (1.7%)	52 (1.6%)	723 (1.9%)
Birth order, n (%)					
First-born	8,468 (49.5%)	5,253 (45.6%)	2,953 (44.8%)	1,470 (44.1%)	18,144 (47.1%)
Second born	6,349 (37.1%)	4,358 (37.8%)	2,487 (37.8%)	1,185 (35.6%)	14,379 (37.3%)
Third or later born	2,298 (13.4%)	1,910 (16.6%)	1,148 (17.4%)	675 (20.3%)	6,031 (15.6%)
Maternal age at birth, n (%)					
<30	5,700 (33.3%)	4,632 (40.2%)	2,818 (42.8%)	1,514 (45.5%)	14,664 (38.0%)
30–34	6,610 (38.6%)	4,205 (36.5%)	2,276 (34.5%)	1,124 (33.8%)	14,215 (36.9%)
≥35	4,805 (28.1%)	2,684 (23.3%)	1,494 (22.7%)	692 (20.8%)	9,675 (25.1%)
Paternal age at birth, n (%)					
<30	4,060 (23.7%)	3,373 (29.3%)	2,076 (31.5%)	1,135 (34.1%)	10,644 (27.6%)
30–34	5,922 (34.6%)	3,925 (34.1%)	2,151 (32.7%)	1,059 (31.8%)	13,057 (33.9%)
≥35	6,892 (40.3%)	4,011 (34.8%)	2,240 (34.0%)	1,055 (31.7%)	14,198 (36.8%)
Missing	241 (1.4%)	212 (1.8%)	121 (1.8%)	81 (2.4%)	655 (1.7%)
Maternal educational attainment, n (%)					
Bachelor's degree or higher	4,913 (28.7%)	2,345 (20.4%)	1,090 (16.5%)	440 (13.2%)	8,788 (22.8%)
Vocational school/junior college graduate	6,098 (35.6%)	4,205 (36.5%)	2,284 (34.7%)	1,100 (33.0%)	13,687 (35.5%)
High school graduate or below	3,935 (23.0%)	3,443 (29.9%)	2,197 (33.3%)	1,227 (36.8%)	10,802 (28.0%)
Missing	2,169 (12.7%)	1,528 (13.3%)	1,017 (15.4%)	563 (16.9%)	5,277 (13.7%)
Paternal educational attainment, n (%)					
Bachelor's degree or higher	7,757 (45.9%)	3,945 (34.8%)	1,862 (28.8%)	818 (25.2%)	14,382 (37.9%)
Vocational school/junior college graduate	2,556 (15.1%)	1,834 (16.2%)	1,067 (16.5%)	538 (16.5%)	5,995 (15.8%)
High school graduate or below	4,422 (26.2%)	4,033 (35.6%)	2,510 (38.9%)	1,333 (41.0%)	12,298 (32.4%)
Missing	2,169 (12.8%)	1,528 (13.5%)	1,017 (15.8%)	563 (17.3%)	5,277 (13.9%)
Maternal smoking at age 6 months, n (%)					
Non-Smoking at 6 months	16,161 (94.4%)	10,690 (92.8%)	5,941 (90.2%)	2,965 (89.0%)	35,757 (92.7%)
Smoking at 6 months	913 (5.3%)	791 (6.9%)	627 (9.5%)	356 (10.7%)	2,687 (7.0%)
Missing	41 (0.2%)	40 (0.3%)	20 (0.3%)	9 (0.3%)	110 (0.3%)
Paternal smoking at age 6 months, n (%)					
Non-smoking at 6 months	10,528 (61.5%)	6,376 (55.3%)	3,384 (51.4%)	1,670 (50.2%)	21,958 (57.0%)
Smoking at 6 months	6,268 (36.6%)	4,866 (42.2%)	3,005 (45.6%)	1,539 (46.2%)	15,678 (40.7%)
Missing	319 (1.9%)	279 (2.4%)	199 (3.0%)	121 (3.6%)	918 (2.4%)
Daycare use at age 1.5 years, n (%)					
Daycare use	11,081 (64.7%)	7,314 (63.5%)	3,871 (58.8%)	1,836 (55.1%)	24,102 (62.5%)
No daycare use	3,889 (22.7%)	2,699 (23.4%)	1,714 (26.0%)	943 (28.3%)	9,245 (24.0%)

Missing	2,145 (12.5%)	1,508 (13.1%)	1,003 (15.2%)	551 (16.5%)	5,207 (13.5%)
Administrative divisions at birth, n (%)					
Special ward or designated city	6,476 (37.8%)	2,521 (21.9%)	1,258 (19.1%)	753 (22.6%)	11,008 (28.6%)
City	9,707 (56.7%)	8,170 (70.9%)	4,472 (67.9%)	2,057 (61.8%)	24,406 (63.3%)
Town or village	932 (5.4%)	830 (7.2%)	858 (13.0%)	520 (15.6%)	3,140 (8.1%)
Major regions, n (%)					
Kanto	9,668 (56.5%)	2,589 (22.5%)	541 (8.2%)	55 (1.7%)	12,853 (33.3%)
Hokkaido	93 (0.5%)	200 (1.7%)	701 (10.6%)	436 (13.1%)	1,430 (3.7%)
Tohoku	383 (2.2%)	753 (6.5%)	1,167 (17.7%)	273 (8.2%)	2,576 (6.7%)
Chubu	3,497 (20.4%)	2,804 (24.3%)	473 (7.2%)	147 (4.4%)	6,921 (18.0%)
Kinki	2,671 (15.6%)	2,074 (18.0%)	1,362 (20.7%)	726 (21.8%)	6,833 (17.7%)
Chugoku	545 (3.2%)	907 (7.9%)	638 (9.7%)	189 (5.7%)	2,279 (5.9%)
Shikoku	19 (0.1%)	353 (3.1%)	524 (8.0%)	183 (5.5%)	1,079 (2.8%)
Kyushu	239 (1.4%)	1,841 (16.0%)	1,182 (17.9%)	1,321 (39.7%)	4,583 (11.9%)

ADI, Area Deprivation Index; Q, quartile.

[a]The uneven distribution of participants across quartiles reflects the actual geographic distribution of births in the cohort relative to municipal-level deprivation.

[b]ADI quartiles were determined based on the distribution of all municipalities in Japan in 2010, where Q1 represents the least deprived areas and Q4 represents the most deprived areas.

eTable 3. Comparison of baseline characteristics between participants who completed the 5.5-year follow-up survey and those lost to follow-up

	Included in analysis (n=24,866)	Loss to follow ups (N=13,688)	All (N=38,554)
Area deprivation index, mean (SD)	5.74 (0.62)	5.83 (0.66)	5.77 (0.63)
Preterm birth <37 weeks, n (%)	1,298 (5.2%)	800 (5.8%)	2,098 (5.4%)
Low birth weight <2,500 g, n (%)	2,303 (9.3%)	1,339 (9.8%)	3,642 (9.4%)
Multiple birth, n (%)	472 (1.9%)	251 (1.8%)	723 (1.9%)
Birth order, n (%)			
First-born	11,988 (48.2%)	6,156 (45.0%)	18,144 (47.1%)
Second born	9,358 (37.6%)	5,021 (36.7%)	14,379 (37.3%)
Third or later born	3,520 (14.2%)	2,511 (18.3%)	6,031 (15.6%)
Maternal age at birth, n (%)			
<30	8,298 (33.4%)	6,366 (46.5%)	14,664 (38.0%)
30–34	9,713 (39.1%)	4,502 (32.9%)	14,215 (36.9%)
≥35	6,855 (27.6%)	2,820 (20.6%)	9,675 (25.1%)
Paternal age at birth, n (%)			
<30	6,011 (24.4%)	4,633 (34.8%)	10,644 (28.1%)
30–34	8,751 (35.6%)	4,306 (32.4%)	13,057 (34.5%)
≥35	9,840 (40.0%)	4,358 (32.8%)	14,198 (37.5%)
Maternal educational attainment, n (%)			
Bachelor's degree or higher	6,990 (29.1%)	1,798 (19.5%)	8,788 (26.4%)
Vocational school/junior college graduate	10,115 (42.1%)	3,572 (38.7%)	13,687 (41.1%)
High school graduate or below	6,942 (28.9%)	3,860 (41.8%)	10,802 (32.5%)
Paternal educational attainment, n (%)			
Bachelor's degree or higher	11,146 (47.0%)	3,244 (36.2%)	14,390 (44.0%)
Vocational school/junior college graduate	4,352 (18.3%)	1,645 (18.3%)	5,997 (18.3%)
High school graduate or below	8,233 (34.7%)	4,082 (45.5%)	12,315 (37.7%)
Maternal smoking at age 6 months, n (%)	1,103 (4.4%)	1,584 (11.6%)	2,687 (7.0%)
Paternal smoking at age 6 months, n (%)	9,211 (37.7%)	6,467 (49.0%)	15,678 (41.7%)
Daycare use at age 1.5 years, n (%)	6,613 (27.5%)	2,632 (28.4%)	9,245 (27.7%)
Administrative divisions at birth, n (%)			
Special ward or designated city	7,277 (29.3%)	3,731 (27.3%)	11,008 (28.6%)
City	15,639 (62.9%)	8,767 (64.0%)	24,406 (63.3%)
Town or village	1,950 (7.8%)	1,190 (8.7%)	3,140 (8.1%)

SD, standard deviation.

eTable 4. Odds ratios for child health outcomes per 1-standard deviation increase of area deprivation index: sensitivity analysis using 80% interval odds ratio (IOR-80%)

	Crude model			Adjusted model[a]		
	OR[b]	IOR-80%		OR[b]	IOR-80%	
Preschool hospitalization						
Any cause	1.04	0.77	1.40	1.04	0.76	1.42
Respiratory infection	1.09	0.74	1.61	1.08	0.73	1.59
Gastrointestinal disease	1.12	0.62	2.01	1.11	0.64	1.93
Kawasaki disease	0.82	0.72	0.94	0.86	0.61	1.21
Asthma	1.10	0.69	1.76	1.10	0.70	1.73
Medical visit						
Preschool asthma	1.03	0.73	1.45	1·05	0.74	1.49
Preschool allergic rhinitis	0.97	0.73	1.29	0.99	0.72	1.37
Preschool atopic dermatitis	0.92	0.73	1.16	0.94	0.82	1.08
Preschool food allergy	0.93	0.66	1.32	1.00	0.73	1.37

Preschool injury	0.96	0.84	1.09		0·99	0.86	1.14	
Intussusception under 2.5 years of age	0.95	0.76	1.86		1·13	0.56	2.27	
Overweight/obesity at 5.5 years of age	1·14	1.12	0.97	1.39		1·11	0.97	1.27

IOR, interval odds ratio; OR, odds ratio.

[a] Adjusted for preterm birth, low birth weight, multiple birth, birth order, mother age category, father age category, mother education, paternal education, maternal smoking, paternal smoking, and municipalities.

[b]The odds ratios presented are posterior median values from Bayesian analysis.

eTable 5. Odds ratios for child health outcomes per 1-standard deviation increase of area deprivation index: sensitivity analysis including only participants who responded to all six longitudinal surveys

		Crude model			Adjusted model[a]		
		OR[b]	95% CrI		OR[b]	95% CrI	
Preschool hospitalization							
	Any cause	1.10	1.05	1.14	1.08	1.05	1.12
	Respiratory infection	1.14	1.08	1.21	1.09	1.05	1.15
	Gastrointestinal disease	1.17	1.07	1.28	1.13	1.05	1.23
	Kawasaki disease	0.95	0.74	1.24	0.89	0.68	1.11
	Asthma	1.16	1.05	1.28	1.08	0.99	1.17
Medical visit							
	Preschool asthma	1.10	1.04	1.15	1.06	1.03	1.10
	Preschool allergic rhinitis	1.04	1.00	1.08	1.02	0.98	1.06
	Preschool atopic dermatitis	0.97	0.93	1.03	0.95	0.91	0.99
	Preschool food allergy	0.98	0.92	1.03	1.00	0.96	1.04

Preschool injury	0.99	0.96	1.03	1.00	0.97	1.04
Intussusception under 2·5 years of age	1.23	0.94	1.56	0.99	0.80	1.20

CrI, credible interval; OR, odds ratio.

[a] Adjusted for preterm birth, low birth weight, multiple birth, birth order, mother age category, father age category, mother education, paternal education, maternal smoking, paternal smoking, and municipalities.

[b] The odds ratios presented are posterior median values from Bayesian analysis.

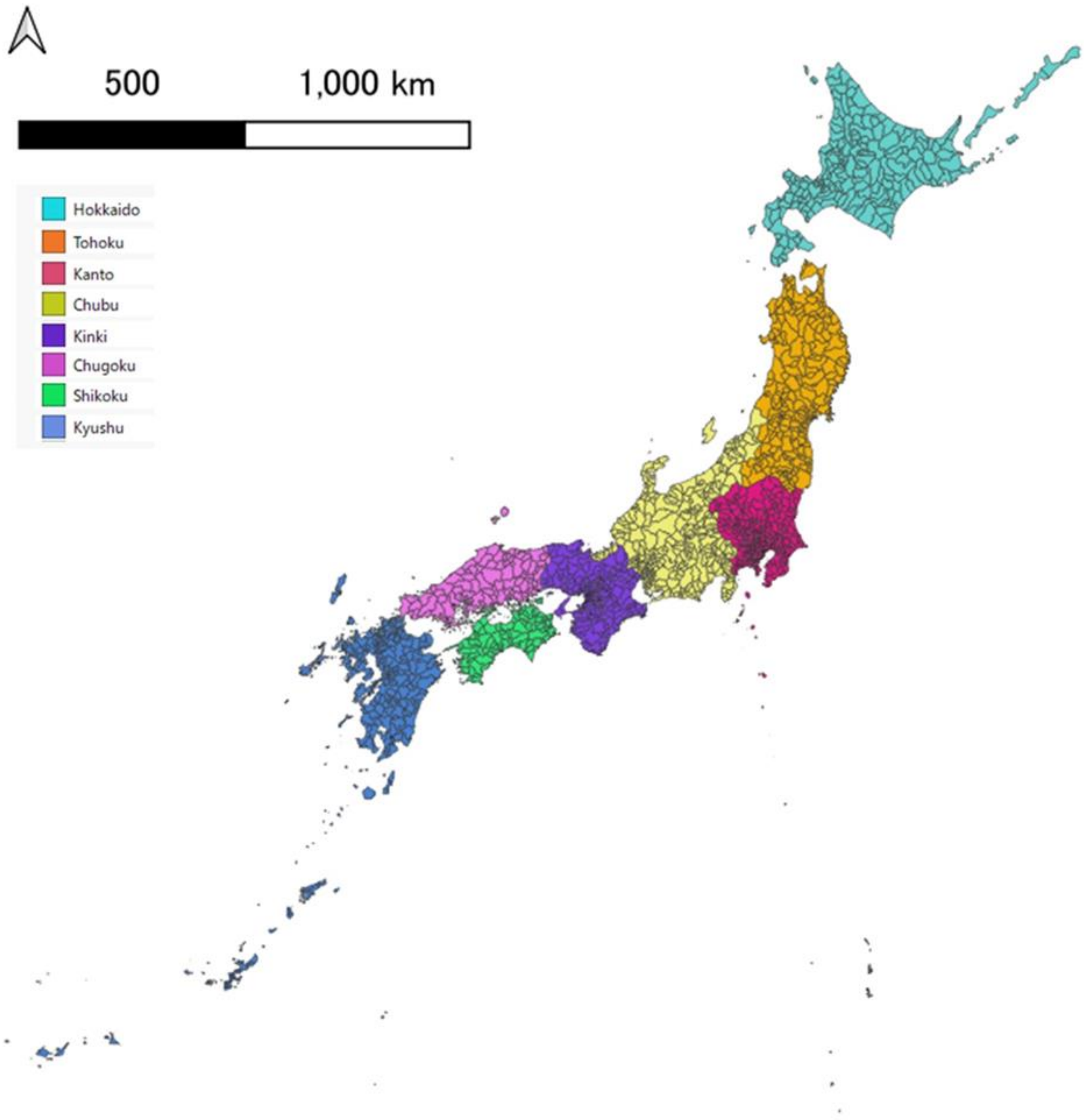

eFigure 1. Distribution of 1,910 municipalities across eight major regions of Japan

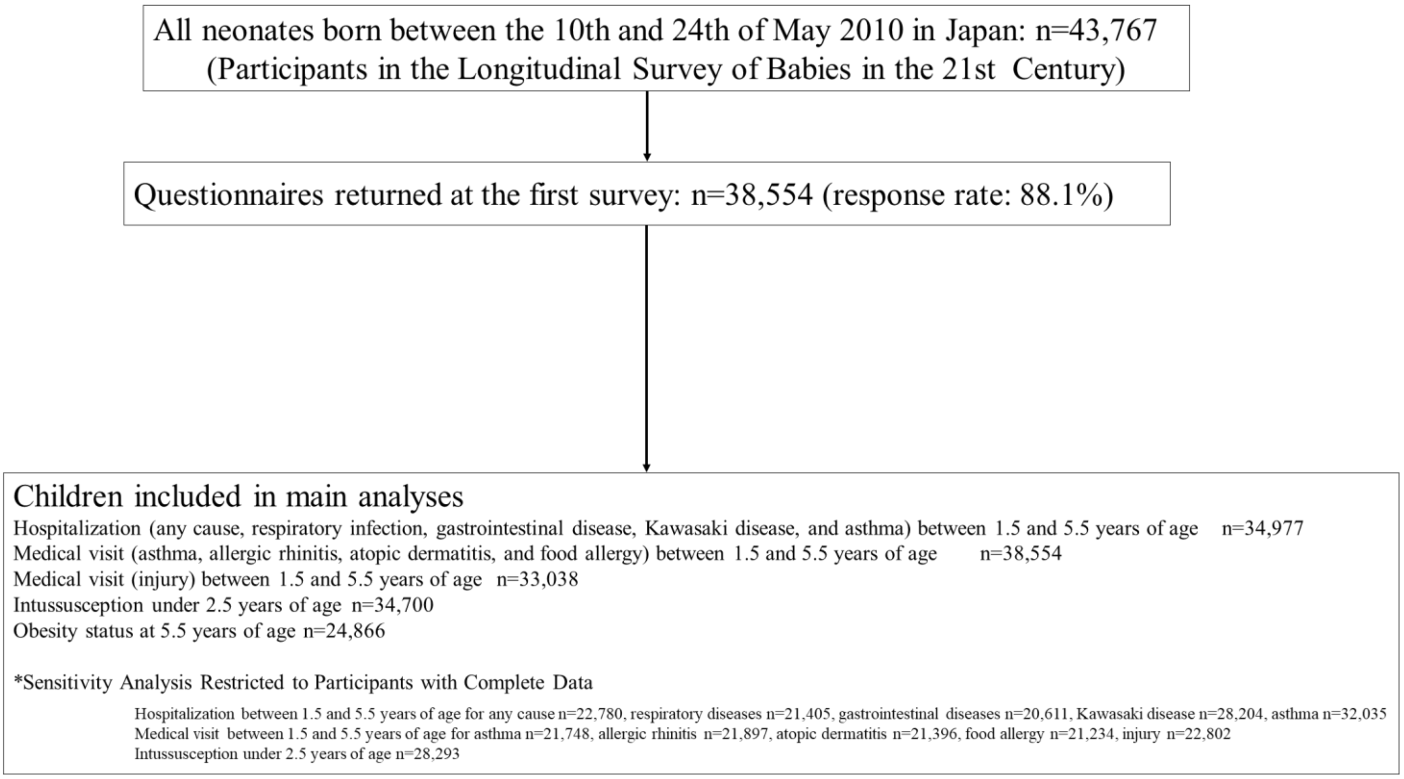

eFigure 2. Flowchart of participants

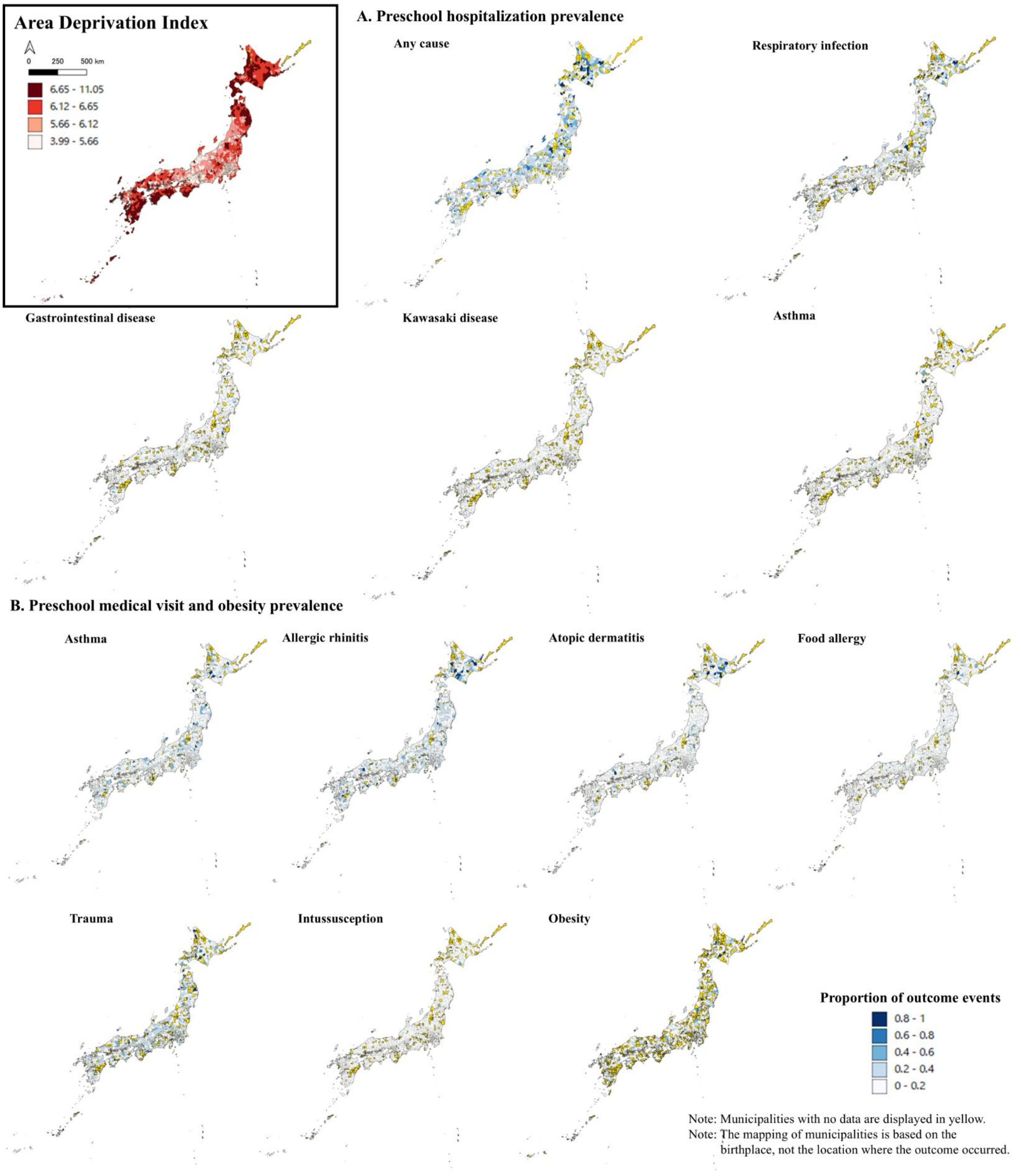

eFigure 3. Geographic distribution of multiple child health outcomes and area deprivation index across Japan
